# New insights in phenotype and treatment of lung disease immuno-deficiency and chromosome breakage syndrome (LICS)

**DOI:** 10.1186/s13023-021-01770-z

**Published:** 2021-03-19

**Authors:** Brigitte W. M. Willemse, Saskia N. van der Crabben, Wilhelmina S. Kerstjens-Frederikse, Wim Timens, Joris M. van Montfrans, Caroline A. Lindemans, Jaap Jan Boelens, Marije P. Hennus, Gijs van Haaften

**Affiliations:** 1 Department of Pediatric Pulmonology and Pediatric Allergology, Beatrix Children’s Hospital, University of Groningen, University Medical Center Groningen, Hanzeplein 1, 9713 GZ Groningen, The Netherlands; 2 Department of Metabolic Diseases, University Medical Center Utrecht, Utrecht, The Netherlands; 3 Department of Clinical Genetics, Amsterdam UMC, University of Amsterdam, Amsterdam, The Netherlands; 4 Department of Genetics, University of Groningen, University Medical Center Groningen, Groningen, The Netherlands; 5 Department of Pathology and Medical Biology, University of Groningen, University Medical Center Groningen, Groningen, The Netherlands; 6 Department of Pediatric Immunology, Wilhelmina Children’s Hospital, University Medical Center Utrecht, Utrecht, The Netherlands; 7 Department of Pediatric Blood and Bone Marrow Transplantation, Princess Maxima Center and UMC Utrecht, Utrecht, The Netherlands; 8 Stem Cell Transplantation and Cellular Therapies Program, Department Pediatrics, Memorial Sloan Kettering Cancer Center, New York, NY USA; 9 Pediatric Intensive Care, Wilhelmina Children’s Hospital, University Medical Center Utrecht, Utrecht, The Netherlands; 10 Department of Genetics (Center for Molecular Medicine, University Medical Center Utrecht (UMCU), Utrecht, The Netherlands

**Keywords:** NSMCE3 gene, Severe respiratory failure, Immunodeficiency, Pediatric acute respiratory distress syndrome (PARDS), Hematopoietic stem-cell transplantation

## Abstract

We report five patients with lung disease immuno-deficiency and chromosome breakage syndrome (LICS) but without recurrent infections and severe immunodeficiency. One patient had extended survival to 6.5 years. Hematopoietic stem-cell transplantation failed to cure another patient. Our findings suggest that the immunological abnormalities can be limited and do not fully explain the LICS phenotype.

## Introduction

The autosomal recessive lung disease immuno-deficiency and chromosome breakage syndrome (LICS), caused by missense mutations in *NSMCE3,* was first described in 2016 [1]. Patients suffered from failure to thrive (FTT), immune deficiency, and fatal pulmonary disease presenting before the age of 2 years. Although it is known that *NSMCE3* is part of the SMC5-SMC6 complex involved in DNA repair, the exact pathophysiology of LICS has not yet been elucidated. It was proposed that disruption of SMC5-SMC6 complex causes a DNA breakage syndrome causing defective T and B cell function. All patients reported so far presented with rapidly progressive irreversible lung damage which seemed to be triggered by (multiple) infection(s).

In this report, we describe the clinical and laboratory characteristics of five recently indentified children with LICS from two unrelated families. Furthermore, we report the outcome of allogenic hematopoietic stem cell transplantation (HSCT) in one of these patients. These findings expand the previously known clinical phenotype and treatment options for LICS. Our findings suggest that testing for *NSMCE3* mutations could be useful in patients with failure to thrive or lung disease of unknown origin.

## Methods

This is a descriptive case series study describing five recently identified LICS patients. Written informed consent following local medical ethics committee guidelines was obtained for all patients. Clinical and laboratory characteristics were obtained from medical records. Genomic DNA was extracted from white blood cells (WBC) from peripheral blood, using standard methods (patient B, her parents and the maternal grandmother in Family 2) or from fibroblasts (patients A, C, D and E).

For patient A, diagnostic exome sequencing was performed in RadboudUMC Nijmegen as previously described [2]. Capture of exons was done using an Agilent SureSelect Human All Exon 50 Mb Kit (Santa Clara, CA, USA). Sequencing was performed using an Illumina Hiseq 2000 (San Diego, CA, USA). Read mapping and variant calling were done using BWA (mapping) and GATK (calling). For patients B, C, D and E, diagnostic sequencing of *NSMCE3* was performed at the University Medical Center Utrecht, Utrecht, The Netherlands [1].

## Results

**Fig. 1:**
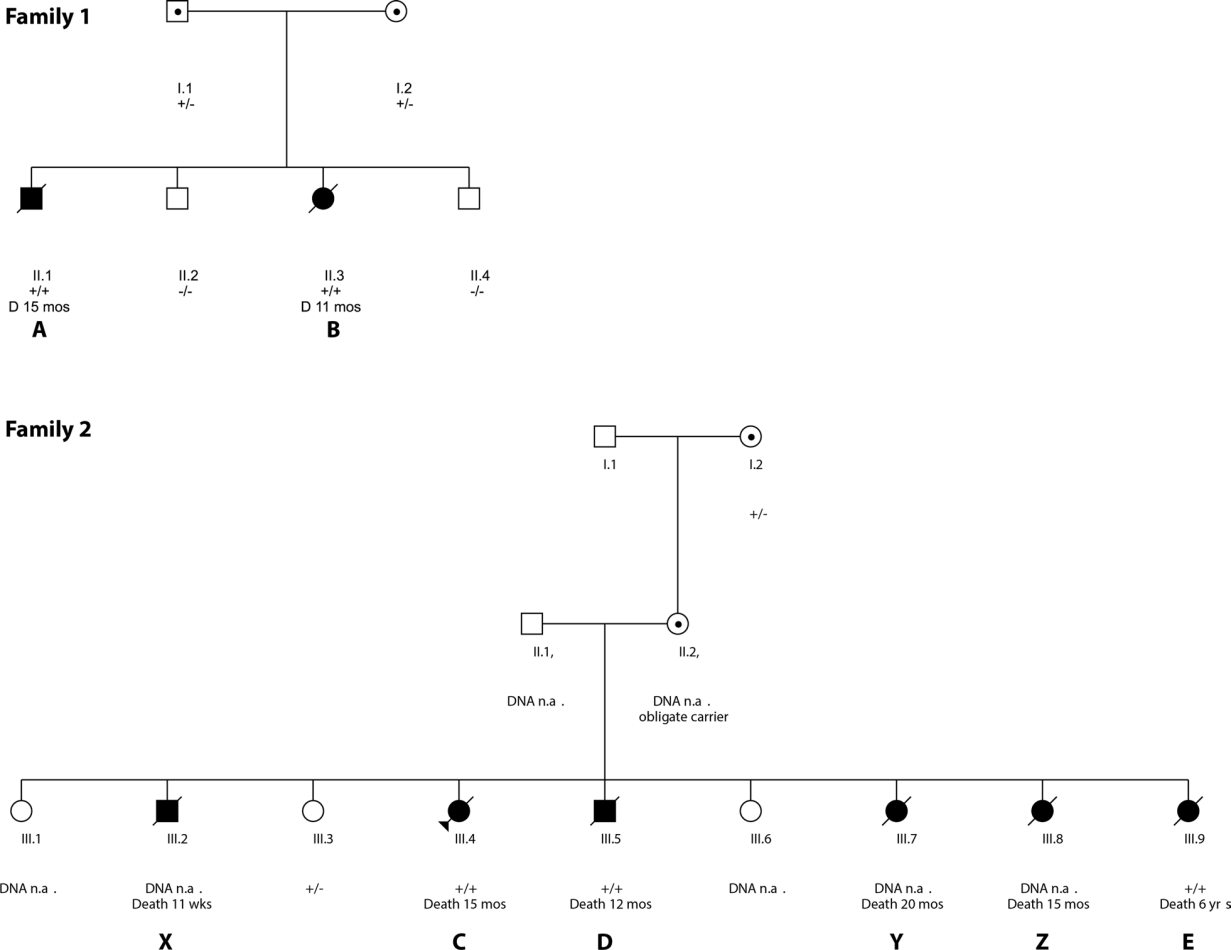
Pedigree of family 1 and family 2. Abbreviations: +/+ homozygous *NSMCE3* (NM_138704.3):c.790C > T, ± heterozygous *NSMCE3* (NM_138704.3):c.790C > T, –/– no mutation in *NSMCE3*. Mos = months, wks = weeks, Capitals refer to patients in main text

All patients (see Table 1 and Table 2 for clinical features) were children of unrelated healthy, non-consanguineous Dutch Caucasian parents (Fig. 1). Four patients (A, C, D and E) were retrospectively identified post mortem after publication of the first paper describing LICS and one patient (B) was diagnosed alive at age 9 months.

**Table 1:** Clinical details of patients A, B, C, D and E affected by LICS syndrome

	Family 1		Family 2		
	A	B	C	D	E
Gender (M/F)	M	F	F	M	F
Age at death (months)	15	11	15	11	78
Mutation *NSMCE3*	Homozygous *NSMCE3* (NM_138704.3):c.790C > T		Homozygous *NSMCE3* (NM_138704.3):c.790C > T		
Common features					
Gestational age (weeks)	38 + 3	37	37 + 3	41	AT
Birth weight (grams)	2450	2240	3100	3380	4000
SGA	+	+	−	−	−
FTT	++	±	++	+	++
Neurological features					
Delayed motor milestones	+	+	+	+	−
Increased infection susceptibility	−	−	−	−	−
Pulmonary features					
Interstitial pneumonia	+	+	+	+	NR
* Viral pneumonia*	−	−	−	−	−
Thymic hypoplasia	−	−	NR	NR	NR
Hypo- or hyperglycemia	NR	Hyper	Hyper	NR	Hypo
Hepato-splenomegaly	NR	+	NR	NR	+

*M *male, *F *female, *AT *at term, *NR* not reported

++present and severe, +present, ±developing, −absent

**Table 2:** immunological features of LICS patients

	Family 1		Family 2		
	A	B	C	D	E*
Immunological features					
IgA (g/L)^a^	2.2 (0.16–1.1)	0.6(0.16–1.1)	1.1(0.16–1.1)	1.0(0.16–1.1)	0.7 (0.19–2.2)
IgG (g/L)^a^	11.1 (2.6–15.2)	7.5 (2.6–15.2)	11.6 (2.6–15.2)	5.3 (2.6–15.2)	8.2 (2.6–15.2)
IgM (g/L)^a^	0.6 (0.1–1.2)	0.6 (0.1–1.2)	2.3 (0.1–1.2)	1.2 (0.1–1.2)	0.7 (0.1–1.2)
IgE (g/L)	344	228	34	273	314
T cells					
CD4 + (10^9^/L)^b^	NA	2.42 (1.7–4.1)	1.12 (1.3–4.1)	28%^c^	1.38 (0.7–2.0)
CD8 + (10^9^/L)^b^	NA	1.06 (0.7–1.8)	0.41 (0.5–1.6)	14%^c^	0.38 (0.5–1.4)
T cell proliferation to					
Mitogens^d^	NA	=	=	NA	↓/=
Antigens^e^	NA	=	NA	NA	=
B cells (10^9^/L)^b^	NA	1.36 (0.8–2.2)	0.68	50%^c^	0.98 *
Antibody titers to					
Diphtheria	NA	=	NA	NA	**
Pneumococcal vaccination	NA	=	NA	NA	**
Delayed type hypersensitivity testing					
Tetanus	NA	=	NA	NA	**
Candida	NA	=	NA	NA	**

= normal, *NA* not available, *at age 2, **not vaccinated

^a^Presented as value (age-matched reference values of serum immunoglobulins)

^b^Presented as value (age-related reference values for lymphocyte subpopulations p10–p90)

^c^Total cells 6 × 10^6^, comment: in lymphoid fraction multiple B-cells, none monoclonal, in T-cell population no abnormalities

^d^Mitogens: Phytohemagglutinin, Concavalin A, Pokeweed

^e^Antigens: Tetanus, candida

**Fig. 2:**
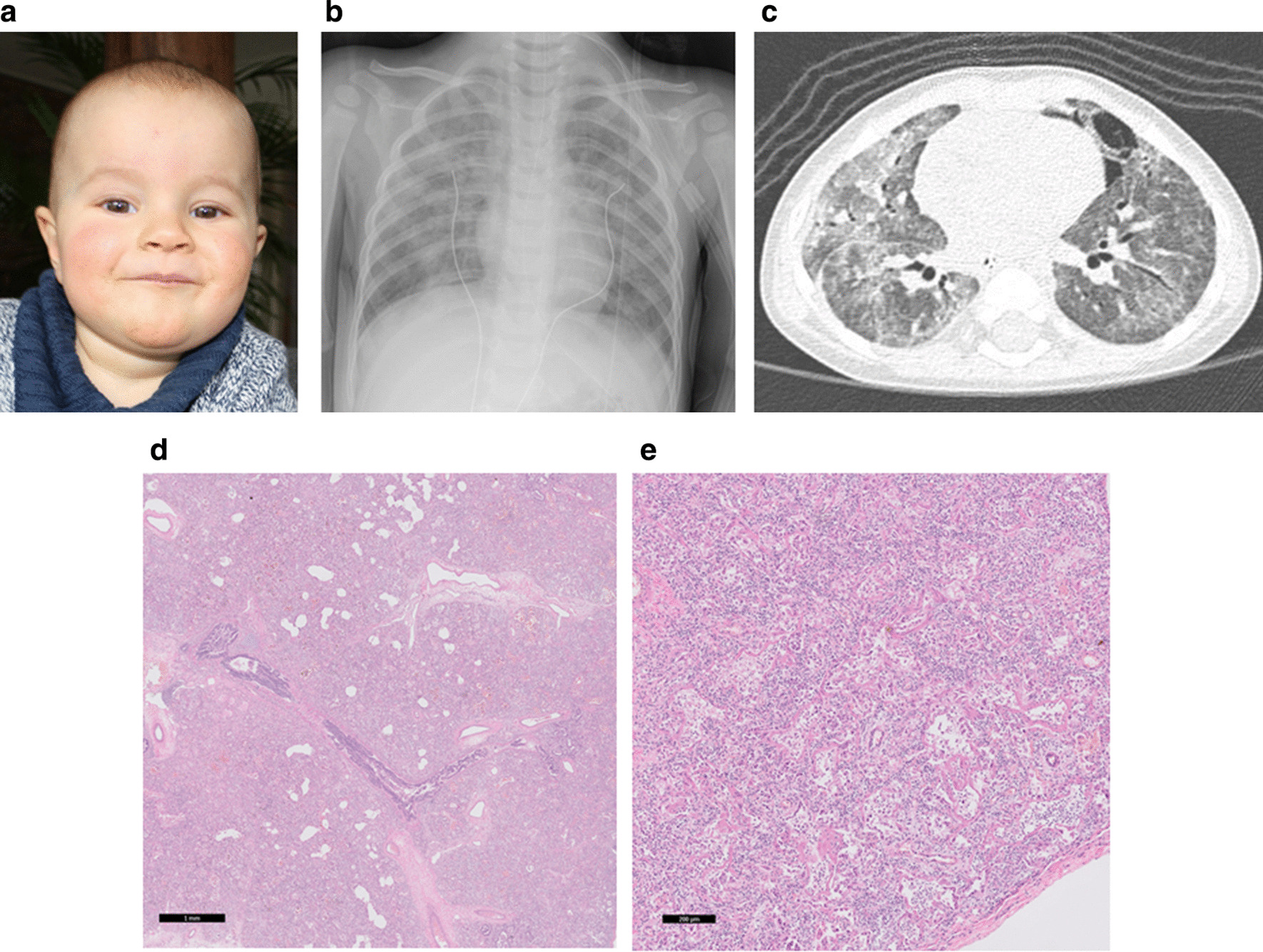
Images of patient A diagnosed with LICS: **a** facial appearance. **b** Chest X-ray on day of admission showing bilateral interstitial infiltrates. **c** corresponding chest CT scan showing bilateral ground glass haziness, interlobar thickening and pneumomediastinum. **e** Lung tissue: overview showing pathological pattern of classic diffuse alveolar damage with extensive thickening of alveolar septa with early interstitial fibrosis (hematoxylin and eosin, scale bar 1 mm). **g** Lung tissue: Magnification: more detailed view, in which remnants of alveoli can be discerned with hyaline membranes, extensive metaplastic squamous alveolar epithelium and widened alveolar septa with variable inflammation and variable stages of fibrosis (hematoxylin and eosin, scale bar 200 micron)

The first male child of Family 1 (patient A) was born small for gestational age (SGA). At the age of 7 months, nasal-gastric tube feeding was started because of FTT. At the age of 13.5 months, he presented with vomiting and tachypnea without signs of infection. He was able to roll over at 7 months and sit stably at 10 months of age. Chest X-ray on admission revealed bilateral diffuse patchy abnormalities and some consolidations, and a CT- scan of the thorax showed severe lung damage with widespread ground glass lesions, thickening of the interlobar septa and pneumomediastinum. No immunological parameters, except immunoglobulins, were studied in patient A, as his clinical course was considered not to be compatible with an immune deficiency. He died at the age of 15 months, of severe respiratory distress despite maximal supportive care and treatment with methylprednisolone. Autopsy showed prolonged diffuse alveolar damage (DAD) as seen in the advanced stage of acute severe interstitial pneumonitis (IP) or pediatric acute respiratory distress syndrome (PARDS) without signs of infection (Fig. 2). Reevaluation of available whole exome sequencing (WES) data, after publication of data on LICS, uncovered a homozygous mutations in *NSMCE3* (NM_138704.3):c.790C > T [1]. The next sibling was a healthy boy, without mutation in the *NSMCE3* gene.

Their younger sister, patient B, born SGA, was clinically evaluated at the age of 8.5 months. After a short period of nasal-gastric tube feeding after birth, she had resumed a normal drinking pattern until shortly before evaluation when decreased intake was noted. She could not roll over and only at only sit for a short period at the age of 9 months. Upon presentation, her respiratory rate was slightly increased. Abdominal ultrasound revealed mild hepatosplenomegaly with homogenous increased density of hepatic and splenic tissue. Karyotyping of peripheral blood lymphocytes showed a normal 46,XX pattern. She had no history of infections and immunological evaluations (Table 2) were normal. Targeted genetic testing revealed homozygosity for the *NSMCE3* (NM_138704.3):c.790C > T mutation.

Because of presumed primary immune deficiency as underlying cause of the initiating events leading to lung pathology in LICS, HSCT was considered a therapeutic option. This thought was based on the results of the previously described children who did show signs of immunodeficiency with low CD4/CD8 T cells and decreased T cell proliferation tests at the time of severe disease. The hypothesis at that time was that the immunodeficiency was responsible for the rapid respiratory deterioration seen in patients with LICS. In retrospect, we have now found more children who did not have major signs of immunodeficiency at time of severe disease. The alternative option of lung transplantation was considered but was not offered, as it proved not to be successful in an earlier patient with LICS. Parents consented and the patient underwent HSCT with unrelated cord blood (10/10 HLA match), after reduced intensity conditioning with fludarabine 150 mg/m2 and cyclophosphamide (cumulative dose of 15 mg/kg), at the age of 10 months. Chimerism increased from an initial 2–3% donor cells, to 60–80% donor cells in the following weeks. The post-transplant course was complicated by sepsis and graft versus host disease, treated with antibiotics and systemic steroids, respectively. After recovery and discharge from the hospital, the patient presented after 3 days with respiratory distress, fever and signs of infection for which she required invasive mechanical ventilation. Chest X-rays showed pneumomediastinum, atelectasis and air entrapment. Chimerism on admission showed 3% donor cells, indicative of rejection of donor stem cells. No treatment options were left and she died at the age of 11 months due to respiratory failure. Post mortem analysis revealed severe DAD with partial organization, presently called acute fibrinoid and organizing pneumonia (AFOP), with iron-laden macrophages without signs of infection.

A fourth and healthy child, was born in Family 1, after prenatal testing for the *NSMCE3* (NM_138704.3):c.790C > T variant showed a wildtype genotype on both alleles.

Family 2 consisted of 9 children. Patients C, D and E suffered from feeding problems mainly vomiting, starting from birth. Patient C had FTT from birth. At the age of 14 months, she was admitted to hospital for gastroenteritis with respiratory distress. At that time, she was able to roll over and sit. Lung biopsy showed IP and extensive DAD. Despite invasive mechanical ventilation and treatment with dexamethasone, respiratory failure was progressive with bilateral pneumothorax and pneumomediastinum. She died 2 weeks after hospitalization. Postmortem DNA analysis revealed homozygosity for the *NSMCE3* (NM_138704.3):c.790C > T mutation.

Patient D, a boy, was evaluated for FTT at 3 months of age. At the age of 10.5 months he was admitted to hospital with respiratory distress requiring invasive mechanical ventilation. At that time, he was able to sit and to roll from back to belly but not back. Chest X-ray on admission showed bilateral pneumothorax, pneumomediastinum and subcutaneous emphysema. Lung biopsy showed severe diffuse IP with DAD. Despite treatment with methylprednisolone he died at the age of 11 months of severe progressive respiratory failure. Postmortem DNA analysis revealed homozygosity for the *NSMCE3* (NM_138704.3):c.790C > T mutation.

Patient E, a girl, was admitted to hospital for reduced intake and hypoglycemia at two years of age. Physical examination showed FTT, mild tachypnea and hepatomegaly. Chest X-ray showed signs of mild interstitial lung disease. During clinical follow-up, she developed signs of respiratory obstruction, e.g. Harrisons grooves and an enlarged anterior–posterior thorax diameter. Motor development was reported as being normal. At 6 years of age, she was admitted to hospital with abdominal pain. Physical findings showed severe FTT, tachypnea and hypoxemia, a prominent sternum, clubbing of fingers and toes, bilateral rales on auscultation of the lungs and severe respiratory distress without signs of respiratory infection. High-resolution-CT-thorax showed bilateral ground glass haziness with diffuse consolidations and mediastinal lymphadenopathy. Treatment with oxygen, methylprednisolone pulse therapy followed by azathioprine, cyclophosphamide and hydroxychloroquine did not result in improvement. Because of previous experience with her siblings, mechanical ventilation was not considered beneficial. She died due to severe respiratory failure two months after admission, at the age of 6.5 years. Postmortem targeted DNA analyses revealed the homozygous mutation in *NSMCE3* (NM_138704.3):c.790C > T.

In this same family patient X, a boy, was born SGA and had FTT from birth. He died unexpectedly at 3 months of age during his sleep. Autopsy findings showed severe IP with enlarged alveolar septa and inflammatory cells consisting of lymphocytes and plasma-cells, without signs of infection. Patients Y and Z, both girls, developed FTT shortly after birth. Patient Z died in hospital at the age of 18 months due to respiratory failure caused by PARDS, despite treatment with prednisolone and chloroquine. Patient Y died at home due to respiratory distress at the age of 15 months. For patients X, Y and Z, either no consent or no material was available for DNA analysis.

During the disease periods where patients suffered from progressive and fatal pulmonary diseases, we found no evidence for macrophage activation syndrome (MAS). None of the patients had high fever or splenomegaly, all had normal hemoglobin, platelets and normal to high values of total lymphocytes. In the children were ferritin was determined, the values were all within the normal range (42–129 mcgr/Liter).

## Discussion

We describe five recently identified patients from two separate families with LICS, all homozygous for one of the previously reported *NSMCE3* mutations. These patients confirmed the previously described phenotype with destructive lung disease at young age, although one patient died at the relatively late age of 6.5 years with signs of chronic respiratory distress. One patient underwent HSCT after reduced intensity conditioning, unfortunately followed by rejection of donor cells and subsequent lung disease with fatal outcome.

Features of early onset FTT, including feeding difficulties and delayed motor milestones were present in all patients except patient E. This patient was the longest surviving patient with LICS reported so far. Her symptoms fitted chronic pulmonary disease, and her cause of death was acute pulmonary failure similar to the other patients. This relatively long survival of a patient with LICS is of clinical importance as it indicates that, apart from the acute and fatal course presenting around 10–14 months, a more chronic form exists as well. We consider it likely that patient X, who suffered from FTT and died suddenly at the age of three months with abnormal pulmonary autopsy findings, may have suffered from LICS as well. This would expand the clinical phenotype to an even earlier presentation, but remains uncertain because no genetic testing could be performed. Until now, 9 patients with LICS were reported. The allele frequency of the *NSMCE3* NM_138704.3):c.790C > T variant in the Dutch population is 0.003, indicating that the variant is not extremely rare in the Netherlands [1]. Mutations in NSMCE3 may be more widespread, and awareness of the disease remains an important issue.

Abnormalities in glucose homeostasis were noted in three patients (patient B, C and E), similar to two previously reported patients [1]. In addition, increased numbers of islets of Langerhans in the pancreas of patient B and in the previously described patients were reported (unpublished data). Hypoglycemia, as noted in several patients might be associated with diminished intake, but could also imply involvement of *NSMCE3*, as part of the SMC5/6 complex in insulin metabolism, as has been previously reported [1, 3]. In addition, in two patients (B and E), hepatomegaly was noted. This had not been reported in previously described patients and might indicate a new, previously unknown feature of this disease with currently unknown etiology.

In other chromosome breakage syndromes, T- and B lymphocyte mediated immune deficiency leading to lung damage is well described [4–6]. In ataxia telangiectasia interstitial lung disease (ILD) has also been suggested as a separate cause of lung damage of unknown etiology [7, 8]. Data on the function of the SMC5/6 complex suggests it plays a role in prevention of transcription from viral circular DNA genomes and that it thereby has a suppressive role in the development of viral infections and inflammatory processes [9–11].

Together with previously published data on LICS patients, HSCT was considered to be possibly curative for the immunodeficiency in LICS, but unfortunately failed. The interpretation of this finding is complicated by the fact that patient B was already mildly symptomatic at the start of the conditioning. An earlier timing may have had a different outcome. Reduced intensity conditioning was given because of the genetic defect and the presumed associated risk for tissue damage secondary to the chemotherapy in the conditioning regimen. After completion of the conditioning regimen, she had no extensive mucositis or other forms of conditioning related tissue damage. The fact that she eventually rejected the donor stem cells shows that her own immunity, even after RIC, was relatively adequate. In contrast to the previously reported patients, none of the patients reported in this paper had recurrent infections. Immunological data in the majority of these patients showed no major immunodeficiency with normal CD4 + and CD8 + T cells numbers and normal T-cell proliferation tests also in contrast to the previously described patients [1]. Altogether, subtle immunodeficiency in B- and T lymphocytes can be part of LICS but is not observed in all patients. We further conclude that immunodeficiency is unlikely to cause the acute respiratory failure in LICS, as this course was also present in the patients with normal immunological evaluations as we now report. Lung transplantation was also shown not to be beneficial in LICS [1], likely because of the underlying systemic nature of this disorder.

With the further confirmation of the pulmonary phenotype in these patients, consisting of PARDS, the pathology findings demonstrating severe DAD with or without inflammatory cells and without signs of infection and the clinical features of FTT, LICS resemble a childhood Interstitial Lung Disease (chILD [12–14]. We propose to categorize LICS as ‘specific condition of undefined etiology’, one of the 9 categories of chILD classification [12].

In conclusion, we present five recently identified LICS patients who developed fatal lung disease without signs of infection and who expressed a variable immunological phenotype. Our findings suggest that the immunological abnormalities can be limited and do not fully explain the LICS phenotype. We further conclude that DNA analysis of *NSMCE3* for LICS should be considered in patients with unexplained chronic pediatric pulmonary disease, in addition to previously reported features of early onset (severe) FTT, (mild) motor developmental delay, and acute pulmonary disease around 10–14 months of age with irreversible DAD. In affected families, genetic counseling and prenatal testing should be offered.

## Data Availability

All data generated or analysed during this study are included in this published article.

## Abbreviations

AFOP: Acute fibrinoid and organizing pneumonia
chILD: Childhood Interstitial Lung Disease
DAD: Diffuse alveolar damage
FFT: Failure to thrive
HSCT: Hematopoietic stem cell transplantation
IP: Interstitial pneumonitis
LICS: Lung disease immuno-deficiency and chromosome breakage syndrome
MAS: Macrophage activation syndrome
PARDS: Pediatric acute respiratory distress syndrome
SGA: Small for gestational age
WES: Whole exome sequencing
WBC: White blood cells
